# Towards Reinforced Brain Tumor Segmentation on MRI Images Based on Temperature Changes on Pathologic Area

**DOI:** 10.1155/2019/1758948

**Published:** 2019-03-03

**Authors:** Abdelmajid Bousselham, Omar Bouattane, Mohamed Youssfi, Abdelhadi Raihani

**Affiliations:** SSDIA Laboratory, ENSET Mohammedia University Hassan 2, Casablanca, Morocco

## Abstract

Brain tumor segmentation is the process of separating the tumor from normal brain tissues; in clinical routine, it provides useful information for diagnosis and treatment planning. However, it is still a challenging task due to the irregular form and confusing boundaries of tumors. Tumor cells thermally represent a heat source; their temperature is high compared to normal brain cells. The main aim of the present paper is to demonstrate that thermal information of brain tumors can be used to reduce false positive and false negative results of segmentation performed in MRI images. Pennes bioheat equation was solved numerically using the finite difference method to simulate the temperature distribution in the brain; Gaussian noises of ±2% were added to the simulated temperatures. Canny edge detector was used to detect tumor contours from the calculated thermal map, as the calculated temperature showed a large gradient in tumor contours. The proposed method is compared to Chan–Vese based level set segmentation method applied to T1 contrast-enhanced and Flair MRI images of brains containing tumors with ground truth. The method is tested in four different phantom patients by considering different tumor volumes and locations and 50 synthetic patients taken from BRATS 2012 and BRATS 2013. The obtained results in all patients showed significant improvement using the proposed method compared to segmentation by level set method with an average of 0.8% of the tumor area and 2.48% of healthy tissue was differentiated using thermal images only. We conclude that tumor contours delineation based on tumor temperature changes can be exploited to reinforce and enhance segmentation algorithms in MRI diagnostic.

## 1. Introduction

A brain tumor represents a set of abnormal cells that reproduce in the brain in an uncontrolled way. There are large varieties of brain tumor types that are classified into two categories, benign (noncancerous) brain tumors are less aggressive, formed slowly, and most often remain isolated from surrounding brain normal tissues; they do not spread to other regions of the brain or other parts in the human body and are generally easier to surgically extract than malignancies. Malignant brain tumors (cancerous) are not always easy to distinguish them from surrounding normal tissues. Therefore, it is sometimes difficult to extract them entirely without damaging the surrounding brain tissues (http://braintumor.org). The number of people affected by malignant brain tumors has been increasing in the last few decades. According to the American cancer society [1, 2] in the US for 2017, there were an estimated number of 23,800 new cases which increased with 30 cases compared to 2016 (23,770) and 16,700 estimated deaths with an increase of 650 cases compared to 2016 (16,050).

Magnetic Resonance Imaging or MRI is a noninvasive medical imaging modality commonly used in the clinical routine as it offers images with high spatial resolution and high contrast between soft tissues. MRI provides rich information about shape, size, and localization of brain tumors for more accurate diagnosis and treatment planning [3, 4]. Therefore, most of the research in medical diagnosis and delineation of brain tumors uses MRI images. Various MRI sequences can be created; they are called weighted images, such as T1-weighted, T2-weighted, Proton-Density Weighted, and Fluid-Attenuated Inversion Recovery (FLAIR). T1-weighted image provides a better segmentation for brain tissues due to the high contrast between gray and white matter [5], T1-weighted contrast-enhanced images and FLAIR are widely used for brain tumors structure diagnostic as it makes tumor region hyperintense. In this work, we have collected synthetic T1-weighted contrast-enhanced and Flair MRI images of all subjects as experimental data to test our approach.

Accurate segmentation of brain tumors from MRI images represents a crucial and challenging task in diagnosis and treatment planning. Image segmentation is an active field in medical imaging, which consists in extracting from the image one or more regions forming the area of interest. Various algorithms have been developed in the literature to perform brain tumor detection, including threshold-based methods [6, 7], region-based methods [8, 9], deformable methods [10–13], classification methods [14, 15], and deep learning [16–18]. Deformable models are among the most popular methods used for brain tumor segmentation in MRI images. They are represented by curves (2D) or surfaces (3D) defined in an image that move by the influence of two forces, internal or local forces defined in the curve to keep it smooth during the deformation process, while external forces are computed from image data in order to move the curve towards the object boundary sought. In the deformable models, we distinguish two principal categories, parametric deformable models or snakes [19] and geometric deformable models. The parametric deformable models necessitate a parametric representation during deformation of the curve. These later have difficulty in topology changes to split and merge contours to segment multiple objects. Geometric deformable models or level sets proposed by Osher and Sethian [20] move based on geometric measurements such as the curve normal and curvature. The advantage of these models is their capacity for topological changes during curve propagation.

Brain tumor segmentation consists of extracting the tumor region from healthy brain tissues; the existence of brain tumors can often be detectable. However, accurate and effective segmentation of tumors remains a challenging task, since the tumors can have different sizes and locations. Their structures are often nonrigid and complex in shape and have various appearance properties. Besides, they have intensities overlapping with normal brain tissues and especially in tumor borders; they show significant variable appearances from patient to patient [21], due to the need to add physical information of tumor to reinforce algorithms segmentation for more accurate and effective extraction. In the present work, we investigate the effect of temperature on segmentation in MRI images. Each tissue in human body has a thermal signature in the presence of abnormality like tumors, the thermal signature of the tissue changes, and the measurement of temperature changes can be helpful for the estimation of the existence and localization of an internal abnormality [22] in recent years was widely used as a tool for tumors diagnostic [23–26]. We have used temperature to delineate tumor contours and compared the obtained results with segmentation by the level set method.

Human body temperature distribution depends on several factors including heat energy generated by cellular metabolism and blood flow, as these are altered in disease; the temperature distribution changes in pathological tissues. The blood flow plays an essential role in the body thermoregulation mechanism, which removes heat from a region with a higher temperature and increases heat in the cooled region. Tumor cells generally generate more heat than adjacent healthy cells due to their high metabolic activity and the blood flow. In the tumorous regions, the blood flow can be significantly less than that in the surrounding healthy tissues [27]. Therefore, heat energy generated by the tumor metabolic heat generation is dissipated less rapidly from the tumor than from the surrounding healthy normal tissues.

Consequently, the tumor temperature rises higher than normal tissues [28]. Kateb et al. [29] showed a significant difference in brain tumor temperature compared to normal brain tissues, up to 3.3°C in the tumor center (36.4°C) compared to the surrounding normal tissues temperature (33.1°C), and demonstrated that it can be used to delineate the margins of brain tumors. Therefore, the temperature distribution can provide additional information about brain tumors. Numerous studies used temperature distribution to detect and estimate tumor size and location using thermal imaging (thermography) [25, 30–33].

In this paper, we developed a new approach to improve the segmentation of brain tumors performance in term of accuracy, based on temperature profiles changes in the tumorous region. The temperature distribution in the brain with the tumor is calculated using Pennes bioheat equation. Next, Canny edge detection method was applied in the calculated thermal image to estimate tumor contours, based on the abrupt change of temperature in tumor contours. The obtained results are compared with Chan–Vese based level set segmentation in MRI images.

The rest of this paper presents the proposed method in Section 2, several tests, results, and discussion in Section 3, and finally, the conclusion in Section 4.

## 2. Materials and Methods

### 2.1. Temperature Calculation

Temperature distribution in the brain with tumors was simulated by Pennes bioheat transfer equation [34], which models heat transfer within biological systems by taking into account heat transfer mechanisms such as thermal conduction, blood perfusion, and metabolic heat generation [34, 35]. This model had some critics in past decades as it does not consider the effect of blood flow direction; it considers that heat equilibration happens in the capillaries and does not take into account the blood leaving the tissue. Several studies tried to overcome these limitations [36–38], but it is still widely used by the majority of papers in the literature due to its implementation simplicity and availability of its parameters experimentally. The Pennes bioheat transfer equation [34, 35] is given by

(1) ρCP∂T∂t=K·∂2T∂x2+∂2T∂y2+ωbρbCpbTa−T+Qm,

where *ρ*  [Kg/m^3^] is the density of tissue, *C*_*P*_ [J / (Kg °C)] is the specific heat of tissue,* K* [W/(m °C)] is the thermal conductivity, *ω*_*b*_ [ml /(s.ml)] is the blood perfusion rate, *ρ*_*b*_ [Kg/m^3^] is the density of blood, *C*_*pb*_ [J/(Kg °C)] is the specific heat of blood, *T*_*a*_ [°C] is the temperature of artery, and *Q*_*m*_ [W/m^3^] is metabolic heat generation. The left-hand side of (1) represents the stored heat energy, the second describes the heat transfer due to conduction; the second term refers to the temperature exchange between the blood and the surrounding tissue, due to blood convection; and the last term denotes the heat generation by cellular metabolism.

To solve (1), normal body temperature *T*_*i*_ = 37°C is considered as the initial condition. In the boundary conditions, brain tissues are supposed to be exposed to constant normal body temperature *T*_*b*_ = 37°C [39]. The thermal properties of blood perfusion were consigned as *ρ*_*b*_= 1052  [Kg/m^3^], *C*_*pb*_= 3800 J/ (Kg. °C), and *T*_*a*_= 37°C [40]. Finite difference method was applied for discretization of Pennes bioheat transfer equation within the Cartesian grid, where i and j represent the pixel index in the image space coordinate. The time step was assumed at Δ*t* = 0.1*s* and the spatial step Δ*x* = Δ*y* = 1  *mm*, which is derived from image resolution. The solver convergence was assumed when the temperature difference within all image pixels between two consecutive iterations is less than 1 · 10^−7  ^. The approximation of the second derivative with respect to both time and space using the finite differences is descripted as follows [41]:

(2) ∂T∂ti,j=Ti,jn+1−Ti,jnΔt,

(3) ∂2T∂x2i,j=Ti−1,j−2Ti,j+Ti+1,jΔx2,

(4) ∂2T∂y2i,j=Ti,j−1−2Ti,j+Ti,j+1Δx2.

After discretization using finite difference method, (1) becomes [42]

(5) Ti,jn+1=Ti,jn+ΔtKρi,jCi,jΔx2·Ti−1,jn+Ti+1,jn+Ti,j−1n+Ti,j+1n−4Ti,jn+Δtρi,jCi,jωbi,jρbi,jCPbi,jTan−Ti,jn+Qi,j,

Table 1 presents the thermal properties used for temperature simulations of normal brain tissues and tumor. A tumor with *ω*_*B*_ = 0.0016  *S*^−1^ and *Q*_*m*_ = 25000  [W/m^3^]  is considered in this study.

Towards the stability and convergence of (5), Δt should satisfy the inequality as follows [42]:

(6) $$\begin{array}{c} \Delta t\leq \frac{2{\Delta x}^{2}\rho C_{P}}{\omega_{b}\rho_{b}C_{pb}{\Delta x}^{2}+12k} \end{array}$$

### 2.2. Chan–Vese Model

Towards the segmentation of brain tumors in T1 contrast and Flair MRI images, we have used active contours without edges proposed by Chan and Vese [45], which is an energy-based method based on the Mumford-Shah segmentation method [46] by approximating the image pixels intensities inside and outside the curve known as c1 and c2, respectively. The minimization problem of energy functional defined by Chan and Vese is described in the following formula:

(7) FCVc1,c2,C=μ∙∫ΩδΦx,y∇Φx,ydxdy+v∙∫ΩHΦx,ydxdy+λ1∫Ωu0x,y−c12HΦx,ydxdy+λ2∫Ωu0x,y−c221−HΦx,ydxdy

where *μ*, *λ*_1_, and *λ*_2_ are positives parameters; in this paper they were initialized at 0.5, 1, and 2, respectively [47]. Φ is level set function, *u*_0_(*x*, *y*) is the input image, C is the curve which corresponding to zero level set function Φ, and H is the Heaviside function [45]:

(8) $$\begin{array}{c} H\left(\;z\;\right)=\left\{\begin{array}{cc} 1, & if\;\;z\geq 0\\ 0, & if\;\;z<0, \end{array}\right. \end{array}$$

and *δ* is one-dimensional Dirac measure [45]:

(9) $$\begin{array}{c} \delta \left(\;z\;\right)=\frac{d}{dz}H\left(\;z\;\right) \end{array}$$

while *ϕ* (x, y) is a signed distance function defined as [45]

(10) Φx,y>0if x,yϵ  InsideCΦx,y=0if x,yϵ  OnCΦx,y<0if x,yϵ  OutsideC

To solve this minimization problem of the energy functional in (7), the gradient descent method is used to derive Euler–Lagrange equations and update the level set functions [45]:

(11) ∂Φ∂t=δΦμdiv∇Φ∇Φ−v−λ1u0−c12+λ2u0−c22

where c1 and c2 are defined as follows [45]:

(12) c1Φ=∫Ωu0x,yHΦx,ydxdy∫ΩHΦx,ydxdyc2Φ=∫Ωu0x,y1−HΦx,ydxdy∫Ω1−HΦx,ydxdy

and we implemented level set method based on the work of Crandall [47].

### 2.3. Canny Edge Detector

The calculated temperature distribution (thermal image) in this study showed that a large gradient in tumor borders is the reason to use an edge detection method to track the tumor contours. An edge in the image represents a strong local variation in pixels intensity, usually, arising on the boundary between two different regions within an image. Edge detection is the process of objects boundaries detection within an image by finding the changes in discontinuities intensities. There are several edge detection methods, developed in the literature. The most famous methods are the edge detection operators of Roberts, Sobel, Prewitt, Kirsh Marr-Hildreth, Robinson, LoG and Canny, and so on. Here, in this work, to detect tumor contours based on temperature distribution, Canny edge detection method [48] was used, as it provides much better results with strong edges compared with the other edge detection methods. Canny is based on a multistage algorithm. It consists of five separate steps: smoothing, gradient finding, nonmaximum suppression, double threshold, and edge tracking using hysteresis. Due to the addition of noise in thermal images, the smoothing step was applied two times.

### 2.4. Segmentation Evaluation

To evaluate the performance of brain tumor segmentation, we have used five metrics, Accuracy, Sensitivity, Specificity, Dice Coefficient, and Jaccard coefficient, which are computed according to the following [49]:

(13) Accuracy=TP+TNTP+FP+TN+FN,

(14) Sensitivity=TPTP+FN

(15) Specificity=TNTN+FP

(16) Dice=2TP2TP+FP+FN

(17) Jaccard=TPTP+FP+FN

where TP “True Positive” counts the number of pixels that are correctly segmented as a tumor and FP “False Positive” represents the number of pixels in the image that are incorrectly segmented as a tumor. FN “False Negative” gives the number of pixels that are incorrectly segmented as healthy pixels, and TN stands for ‘‘True Positive” denotes the number of pixels that are correctly segmented as healthy pixels.

### 2.5. Experiments on Synthetic MRI Images

**Figure 1:**
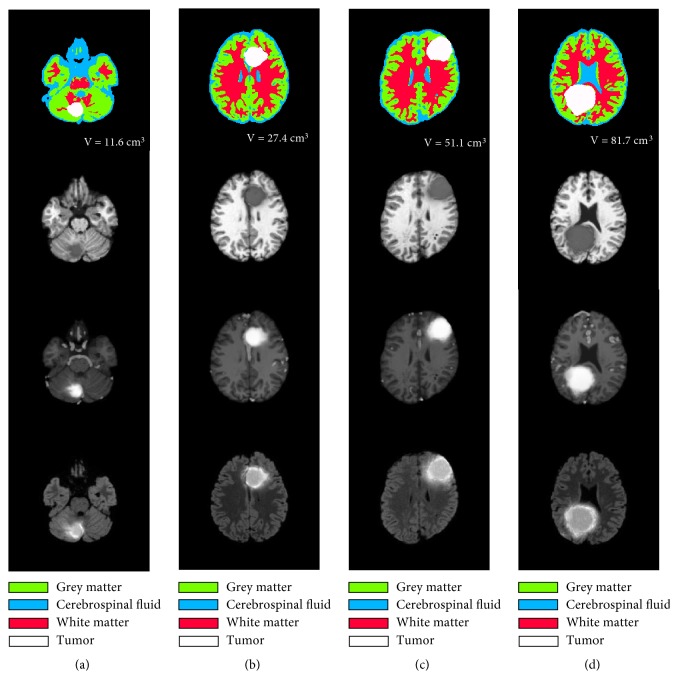
Synthetic T1, T1 contrast, and Flair images of four patients with tumors of different volumes with their ground truth. (a) Tumor with 11.6 cm^3^ of volume. (b) Tumor with 27.4 cm^3^ of volume. (c) Tumor with 51.1 cm^3^ of volume. (d) Tumor with 81.7 cm^3^ of volume.

To validate the approach in tumors with different locations and volumes, we have taken four synthetic MRI images of patients with brain tumors from [50], with 11.6  *cm*^3^ for patient 1, 27.4  *cm*^3^ for patient 2, 51.1  *cm*^3^ for patient 3, and 81.7  *cm*^3^ for patient 4. The tumors were generated using realistic 3D tumor growth cross-platform software called TumorSim simulator [51]. It was used to validate brain tumor segmentation in many recent papers [52–54]. The database containing 100 MRI images of brains with tumors of different locations and volumes was created from 20 patients in the BrainWeb database, five images per patient [55]. Each generated MR image includes 181 slices.  Figure 1 shows that the T1, T1 contrast-enhanced, and FLAIR slices were taken from four different patients with different tumor volumes in the axial plane associated with its ground truth. The 2D axial planes are presented for varying brain patients. Each one of the slices is taken at the axial plane of maximum tumor surface. All the images are 256 x 256 pixels, 12–bit grayscale in DICOM format, and have 1 mm^2^ isotropic resolution.

Fifty other synthetic patients were used to test our approach; 25 patients with high-grade tumors were taken from BRATS 2012 Training data, 25 other patients with low-grade tumors were taken from BRATS 2013 Training data [56, 57]; these synthetic images were created using TumorSim [51]. The reason of validating the approach in synthetic MRI images is that it contains the ground truth of brain tissues and tumors; therefore, we take the real geometries of tissues and tumors for more accurate temperature calculation.

The bioheat transfer equation, level set method, and Canny edge detection method were implemented using C/C++ on Windows 7 operating system with a CPU Intel i7-4770k. The C/C++ code has been compiled with Visual C++ compiler. The DICOM images were read using ITK (www.itk.org) library.

## 3. Results and Discussion

**Figure 2:**
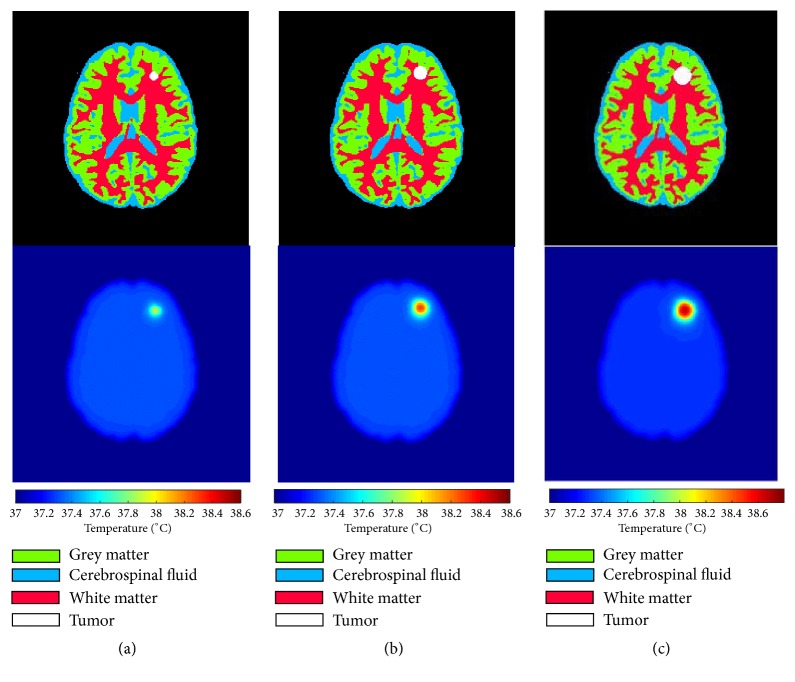
Temperature distribution of brain with circular tumors of three different diameters. (a) Tumor with 10 mm of diameter. (b) Tumor with 15 mm of diameter. (c) Tumor with 20 mm of diameter.

**Figure 3:**
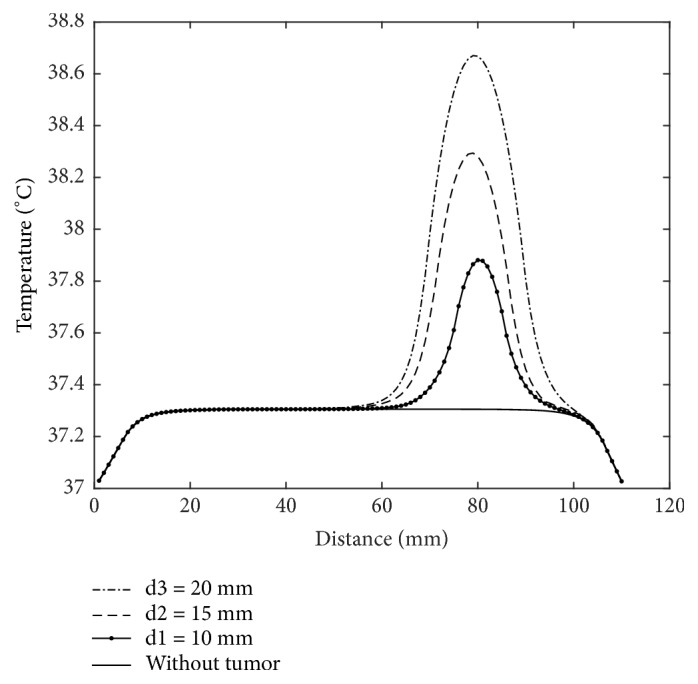
1D representation of temperature profile on the path passes through the tumors centers with different sizes.

Brain tumor thermally represents a heat source; its volume affects temperature distribution. A simplified circular tumor with three diameters is placed in the same location in the healthy brain as shown in Figure 2. At first glance, we can observe that temperature increases in tumors with higher sizes (volume). For tumors with 10 mm, 15 mm, and 20 mm the temperature increases with 0,58°C, 0,99°C, and 1,37°C, respectively, where the maximum temperature is in the tumor center.  Figure 3 provides more clear representation of 1D temperature profile in the line passing through the center of tumors. Results show clearly the existence of abnormality; also, the rises in temperature distribution do not only indicate the existence of a tumor but also provide useful information about its localization.

**Figure 4:**
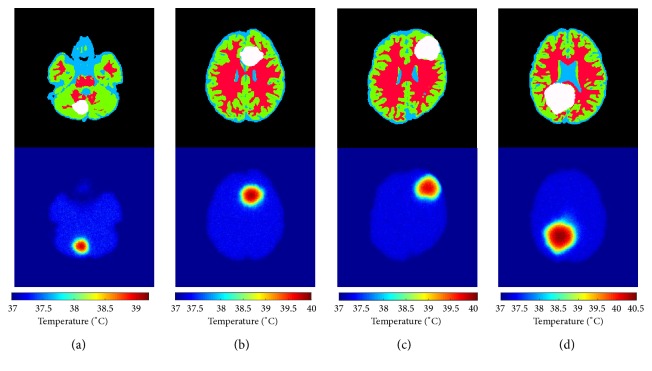
Temperature distribution with noise of brains with realistic tumors of different volumes. (a) Tumor with 11.6 cm^3^ of volume. (b) Tumor with 27.4 cm^3^ of volume. (c) Tumor with 51.1 cm^3^ of volume. (d) Tumor with 81.7 cm^3^ of volume.

Next, the temperatures distributions of the brain with realistic tumors were calculated with the addition of Gaussian noise; Figure 4 presents the synthetic images used for the analysis of the proposed approach; it gives the ground truth of four patients with different tumor volumes in different locations and illustrates the corresponding calculated temperature distribution. The maximum temperatures rise which is in the center of tumors with volumes of 11.6* cm*^3^, 27.4  *cm*^3^, 51.1  *cm*^3^, and 81.7  *cm*^3^ being 1.86°C, 2.53°C, 2.75°C, and 3.12°C, respectively without addition of noise. The obtained results confirm that temperature is high with increasing tumor volumes.

**Figure 5:**
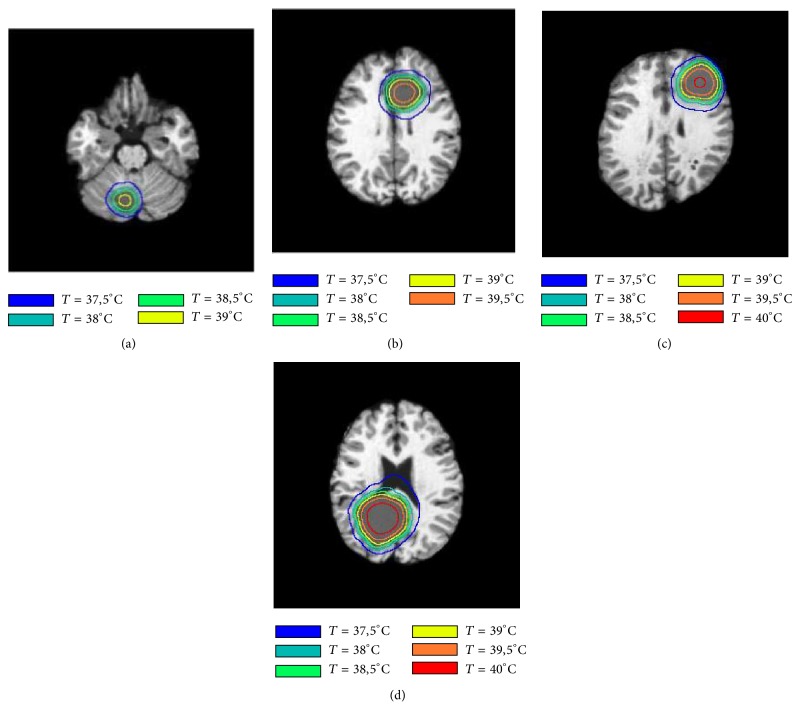
Temperature isotherms in the four cases to show the degree of variation of temperature in the tumorous region.

**Figure 6:**
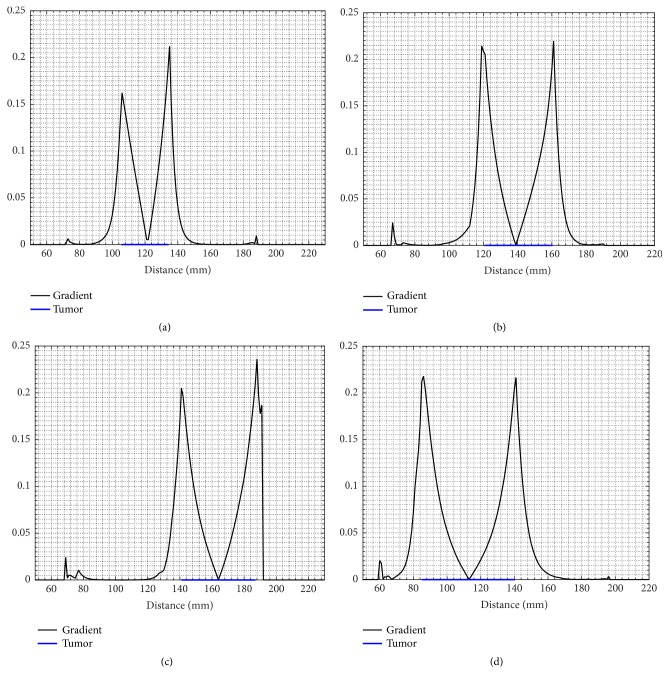
1D representation of temperature absolute gradient on the path passes in the tumor center in the four cases. (a) Tumor with 11.6 cm3 of volume. (b) Tumor with 27.4 cm3 of volume. (c) Tumor with 51.1 cm3 of volume. (d) Tumor with 81.7 cm3 of volume.

Figure 5 shows drawn temperature isotherm without noise on T1-weighted images based on tumor temperature profile to analyze the degree of variation of temperature in the tumorous region. Six curves in different colors with 0.5°C of difference between each curve were drawn to represent temperature lines of 37.5°C, 38°C, 38.5°C, 39°C, 39.5°C, and 40°C, respectively. For the four patients, the curve with 37.5°C of temperature represents healthy pixels. However, these pixels are affected by tumor temperature. Also, as the tumor volume increases this curve moves away from the tumor borders, as shown in Figures 2, and 4 tumors with high volume generate more heat compared to tumors with fewer volumes. Notice that not only the temperature increases but also it has a larger distribution; this explains that curve with 37.5°C is located far from contours in tumors of high volume. We also observe that temperature has an abrupt change in the tumor contours compared to tumor core and the healthy area; this observation is confirmed in Figure 6, which shows a 1D absolute gradient in the line passing across the tumor center. It can be inferred that the temperature gradient always has the maximum value in the tumor contours for all the four patients, which proves that tumor thermal profile can provide rich information about tumor borders that can be used to reinforce segmentation algorithms. This assumption represents the basis on which our approach has been developed using Canny edge detection method to detect tumor contours from temperature distribution.

**Figure 7:**
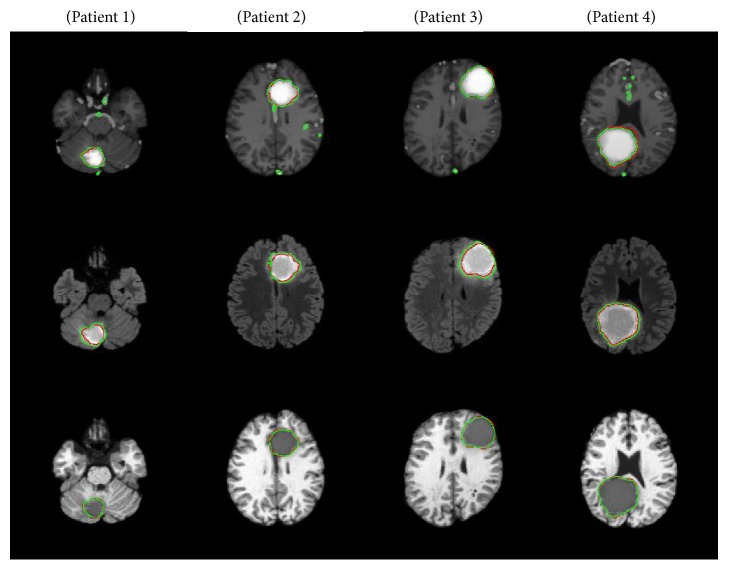
Results of segmentation by level set method in MRI images and the proposed approach. The first and second lines provide the segmentation by level set in T1 contrast and Flair respectively. The last line gives the segmentation using the proposed approach showed in T1. Green: segmentation. Red: ground truth.

The results of the segmentation are illustrated in Figure 7, where green and red curves represent segmentation and ground truth, respectively. Each patient is presented in different column; the two first lines present the results of segmentation using the level set method on different MRI sequences, which are T1 contrast and Flair, respectively. The last line gives the obtained results of segmentation using the proposed approach based on Canny edge detection from temperature distribution with the addition of Gaussian noise shown in T1-weighted images. At first glance, it can be inferred that segmentation has been improved significantly using the proposed approach. These results demonstrate that temperature provides rich information about tumor margins that can be exploited to have more effective tumor segmentation in MRI images.

To evaluate the segmentation performance, we have used five metrics, Accuracy, Sensitivity, Specificity, Dice Coefficient, and the Jaccard coefficient. The obtained results are presented in Table 2; it presents the calculated segmentation evaluation metrics for level set method in different MRI sequences and the proposed approach. The proposed approach yields good results compared with level set segmentation for the four patients. All the metrics were improved in all cases, except Sensitivity, which is reduced. It can be justified by the fact that, in the segmentation in MRI using level set, the number of FN is very weak and sometimes null, as most of the curve of the level set remains outside the tumorous area.

**Figure 8:**
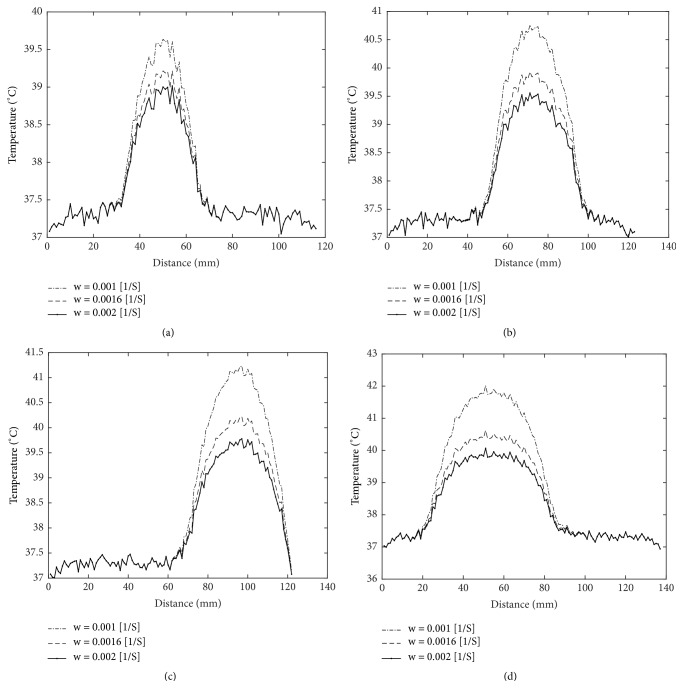
Temperature distribution with noise of brains with realistic tumors of different volumes by considering three values of blood perfusion rate. (a) Tumor with 11.6 cm^3^ of volume. (b) Tumor with 27.4 cm^3^ of volume. (c) Tumor with 51.1 cm^3^ of volume. (d) Tumor with 81.7 cm^3^ of volume.

The temperature is obtained using the Pennes bioheat equation, which can produce errors in calculation compared to the experimentally measured temperature, as the model is isotropic and the used thermal properties do not represent the realistic properties of the patient. However, our interest in this work is the way the temperature is diffused, and its variation in tumor borders independently of the degree of temperature rises in the tumorous region.  Figure 8 shows a one-dimension temperature distribution with noise in the tumorous regions by considering three different values of blood perfusion rate which are 0.001  *S*^−1^  , 0.0016  *S*^−1^, and 0.002  *S*^−1^  for the same tumor.  Table 3 furnishes the calculated segmentation evaluation metrics for the proposed approach using Canny method applied on three temperature profiles of the same tumor for all patients. It can be inferred that the obtained results of segmentation are with good accuracy for all cases. In addition, there is no significant difference by applying the approach on three tumor temperature profiles of the same tumor, as there is a high variation of temperature in tumor borders in the three cases, which proves more the feasibility and the robustness of the proposed approach.

Thus far, tumors contours were detected using steady-state thermal analysis, where the segmentation was performed in the equilibrium state of temperature distribution. In order to study the effect of transient thermal analysis in brain tumors segmentation, cold stress was applied. From an initial temperature distribution at thermal equilibrium obtained using (5), a cold stress temperature *T*_*cold*_ was reducing from each pixel temperature. We have considered three values for *T*_*cold*_, 0.25°C, 0.5°C, and 1°C. After cooling the brain, (5) was then solved. Next, thermal images were obtained at different time steps, 5 s, 100 s, 600 s 1000 s, 2000 s, and 2500 s, respectively.  Table 4 depicts the obtained results of segmentation evaluation of the obtained thermal images at each time step. It can be observed that, in the three cases for all times steps, the approach is still giving acceptable results, which shows the applicability of the approach even in transient temperature distribution.

**Figure 9:**
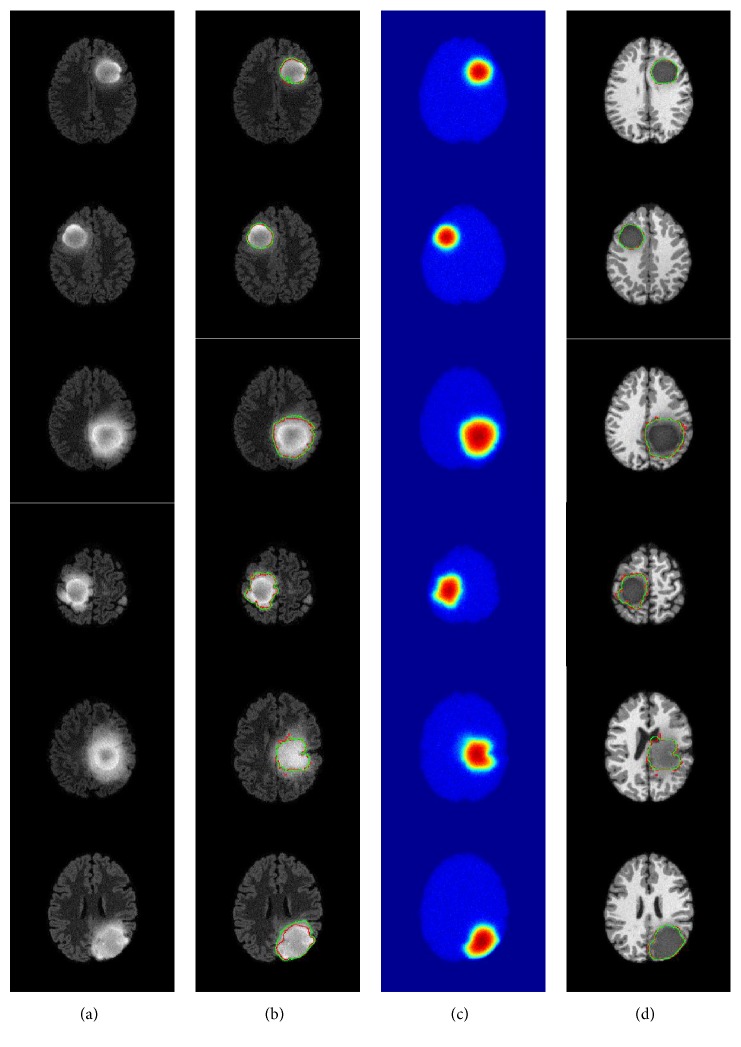
Results of segmentation by level set method in Flair MRI images and the proposed approach applied in six patients taken from BRATS 2012. (a) Flair images (b) segmentation by level set in Flair images. (c) Temperature distribution with noise (d) the segmentation using the proposed approach showed in T1-weighted images (green: segmentation, red: ground truth).

**Figure 10:**
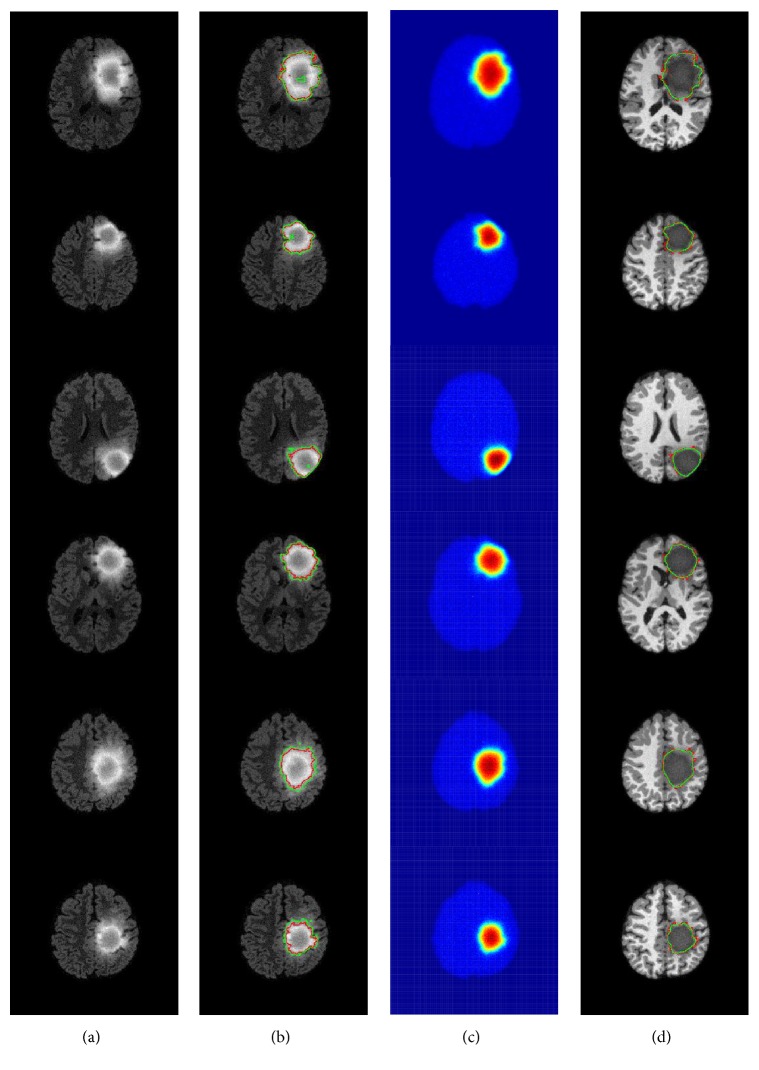
Results of segmentation by level set method in, MRI images and the proposed approach applied in six patients taken from BRATS 2013. (a) Flair images (c) segmentation by level set in Flair images. (c) Temperature distribution with noise (d) the segmentation using the proposed approach showed in T1-weighted images (green: segmentation, red: ground truth).

Figures 9 and 10 depict the results of segmentation of level set in Flair images and the proposed approach in the thermal images with additional noise of fifty synthetic patients taken from BRATS 2012 Training data and BRATS Training data, respectively. Tables 5 and 6 report the performance evaluation of the 50 patients. In all tested cases, the delineation of tumor contours based on temperature distribution showed a significant improvement compared to the level set method, except Sensitivity and the reason is explained in the previous case.  Table 7 presents the percent of the tumor and healthy areas differentiated by segmentation in thermal images only in all test cases; from the two tables, it can be observed that segmentation is reinforced using thermal images, which lead to more effective segmentation of brain tumors.

In Figure 9 and Table 5, patient 4 and patient 5 show fewer values of the used segmentation evaluation metrics (Dice, Jaccard…, etc.) using the proposed approach; this assumption is also observed in Figure 10 and Table 6 for patients 2 and patient 6. This can be explained as the temperature is calculated using the standard Pennes equation, which is isotropic model and biological tissues are highly anisotropic. Accordingly, the edge detected by Canny method is smooth and finds difficulties in estimating complex geometries. In future works, we plan to modify the standard Pennes equation to consider anisotropy using MRI Diffusion Tensor Imaging (DTI) to guide the anisotropy, in order to obtain more accurate and realistic temperature distribution in the tumorous region. Next, we will develop a method to apply the proposed approach in clinical realistic MRI images and use the obtained results to reinforce other recent methods in the literature to prove more the effectiveness of the proposed approach.

**Figure 11:**
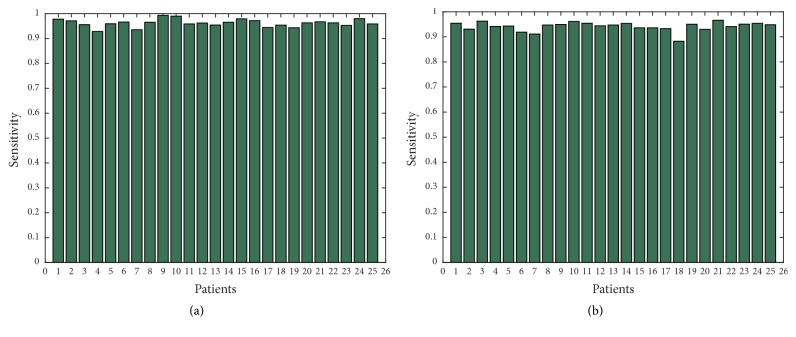
Sensitivity in thermal images for 50 patients; (a) 25 with high-grade taken from BRATS 2012; (b) 25 with low-grade taken from BRATS 2013.

**Figure 12:**
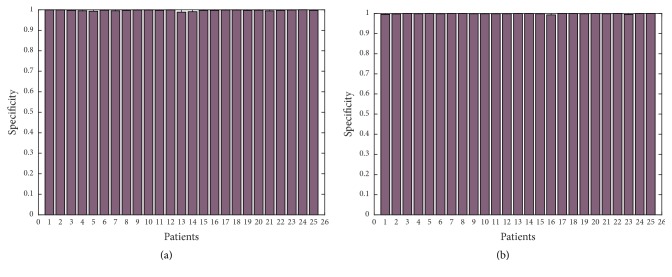
Specificity in thermal images for 50 patients; (a) 25 with high-grade taken from BRATS 2012; (b) 25 with low-grade taken from BRATS 2013.

**Figure 13:**
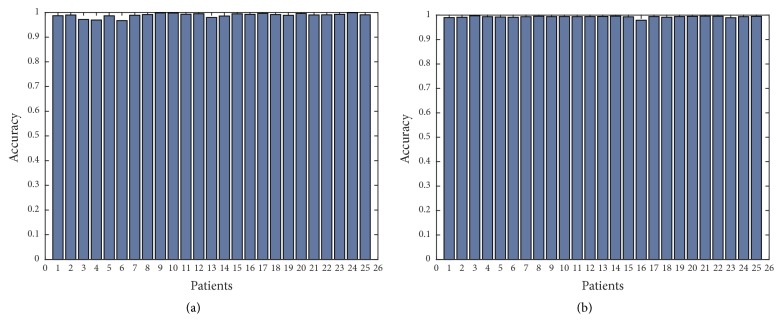
Accuracy in thermal images for 50 patients; (a) 25 with high-grade taken from BRATS 2012; (a) 25 with low-grade taken from BRATS 2013.

**Figure 14:**
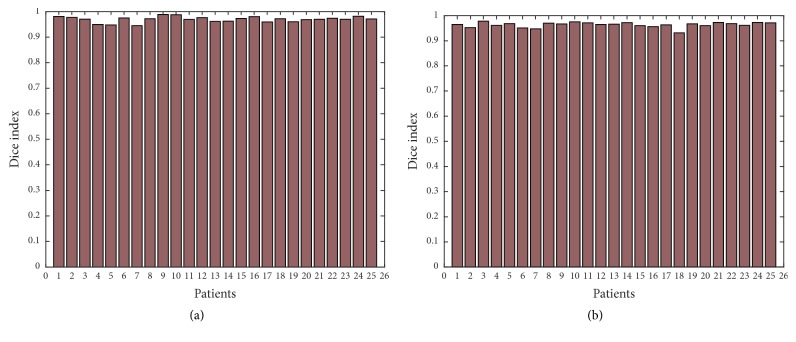
Dice index in thermal images for 50 patients; (a) 25 with high-grade taken from BRATS 2012; (b) 25 with low-grade taken from BRATS 2013.

**Figure 15:**
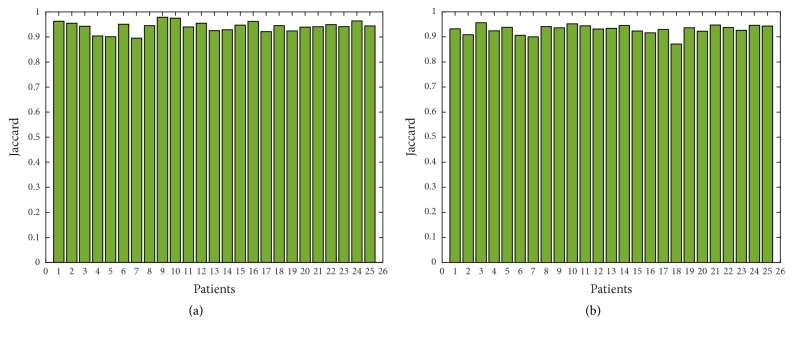
Jaccard in thermal images for 50 patients; (a) 25 with high-grade taken from BRATS 2012; (b) 25 with low-grade taken from BRATS 2013.

In order to show further the robustness of the proposed approach, we have applied Canny edge detector in obtained thermal images with additional noise of all 25 patients with high-grade tumors taken from BRATS 2012 database and 25 patients with low-grade tumors taken from BRATS 2013 database. Figures 11, 12, 13, 14, and 15 show the obtained results of Sensitivity, Specificity, Accuracy, Dice index, and Jaccard, respectively, for all patients. In all the tested cases, the estimation of tumor contours was accurate.

In this work, we considered temperature distribution for brain tumor borders delineation. Brain tumors modify the normal temperature due to the variation in heat generation by cells metabolism and blood flow in tumors. Temperature reveals abrupt changes in tumor borders. Thus, we used the Canny edge detection method to locate the edges. The experiments showed that the proposed approach detects tumor borders with good accuracy and reduces false positive and false negative of segmentation by level set method in MRI images used in clinical routine. To the best of our knowledge, we are the first to incorporated thermal analysis of brain tumor in MRI images segmentation.

## 4. Conclusions

Effective and accurate brain tumor segmentation from MRI images is still a challenging task due to the structural complexity of brain tumors. In this paper, we proposed a new approach to enhance brain tumor segmentation based on the thermal analysis of brain tumors. We have presented and investigated the effect of tumor on brain temperature distribution as well as its size on temperature distribution. Next, we have used tumor thermal profile for segmentation to detect tumors contours. We calculated the temperature distribution in the brain using Pennes bioheat equation implemented by finite difference method (FDM). The obtained results were compared with level set method tested in different synthetic MRI sequences of different patients. We showed a significant improvement in segmentation accuracy. Therefore, the proposed approach can be used as a new indicator to enhance tumors segmentation. The present work can be very useful towards the creation of a new MRI thermal imaging sequence in future studies, which measure the absolute temperature distribution, as all MR-based temperature-mapping approaches require a baseline data set.

## Figures and Tables

**Table 1:** Thermal properties used for temperature simulation.

Material	Property name					
	*k *[*W*/(*m*°*C*)]	*ρ*[*kg*/*m*^3^]	*C*_*p*_[*J*/(*Kg* °*C*)]	*Q*_*m*_[*W*/*m*^3^]	*ω*_*b*_[*ml*/(*ml*•*s*)]	Refs
CSF	0.6	1000	4200	0	0	[43]
GM	0.565	1035.5	3680	16,229	0.013289	[43]
WM	0.503	1027.4	3600	4517.9	0.0036956	[43]
Tumor	0.565	1027.4	3600	25,000	0.0005	[31, 43, 44]

**Table 2:** The calculated segmentation evaluation metrics for level set method and proposed approach.

Patient No.	Method	*TP*	*FP*	*TN*	*FN*	*Sensitivity*	*Specificity*	*Accuracy*	*Dice index*	*Jaccard*
Patient 1	T1c	593	191	14609	0	1	0.987	0.9875	0.8612	0.7563
	Flair	592	285	14515	1	0.9983	0.9807	0.9814	0.8054	0.6742
	*Thermal map*	*556*	*5*	*14795*	*37*	*0.9376*	*0.9996*	*0.9972*	*0.9636*	*0.9297*

Patient 2	T1c	1160	287	18241	0	1	0.9845	0.9854	0.8899	0.8016
	Flair	1138	369	18159	22	0.981	0.98	0.9801	0.8533	0.7442
	*Thermal map*	*1112*	*37*	*18491*	*48*	*0.9586*	*0.998*	*0.9956*	*0.9631*	*0.9289*

Patient 3	T1c	1593	105	18116	28	0.9827	0.9942	0.9932	0.9599	0.9229
	Flair	1598	431	17790	23	0.9858	0.9763	0.9771	0.8756	0.7787
	*Thermal map*	*1547*	*7*	*18214*	*74*	*0.9543*	*0.9996*	*0.9959*	*0.9744*	*0.9502*

Patient 4	T1c	2224	109	17545	204	0.9159	0.9938	0.9844	0.9342	0.8766
	Flair	2428	418	17236	0	1	0.9763	0.9791	0.9207	0.8531
	*Thermal map*	*2375*	*25*	*17629*	*53*	*0.9781*	*0.9985*	*0.9961*	*0.9838*	*0.9682*

**Table 3:** The calculated segmentation evaluation metrics for the proposed approach by considering different values of blood perfusion rate.

Patient No.	*ω*_*b*_	*TP*	*FP*	*TN*	*FN*	*Sensitivity*	*Specificity*	*Accuracy*	*Dice index*	*Jaccard*
Patient 1	0.001	549	3	14797	44	0.9258	0.9997	0.9969	0.9589	0.9211
	0.0016	556	5	14795	37	0.9376	0.9996	0.9972	0.9636	0.9297
	0.002	559	5	14795	34	0.9426	0.9996	0.9974	0.9662	0.9347

Patient 2	0.001	1099	29	18499	61	0.9474	0.9984	0.9954	0.9606	0.9243
	0.0016	1112	37	18491	48	0.9586	0.998	0.9956	0.9631	0.9289
	0.002	1118	47	18481	42	0.9637	0.9974	0.9954	0.9617	0.9262

Patient 3	0.001	1538	1	18220	83	0.9487	0.9999	0.9957	0.9734	0.9482
	0.0016	1547	7	18214	74	0.9543	0.9996	0.9959	0.9744	0.9502
	0.002	1558	14	18207	63	0.9611	0.9992	0.9961	0.9758	0.9529

Patient 4	0.001	2330	17	17637	98	0.9596	0.999	0.9942	0.9759	0.9529
	0.0016	2375	25	17629	53	0.9781	0.9985	0.9961	0.9838	0.9682
	0.002	2387	31	17623	41	0.9831	0.9982	0.9964	0.9851	0.9707

**Table 4:** The calculated segmentation evaluation metrics for transient thermal analysis in brain tumor contours detection.

T_*cold*_ (°C)	Time (s)	*Sensitivity*	*Specificity*	*Accuracy*	*Dice index*	*Jaccard*
*0.25*	5	0.9773	0.9986	0.996	0.9836	0.9677
	100	0.9761	0.999	0.9963	0.9846	0.9697
	600	0.9794	0.9985	0.9962	0.9842	0.969
	1000	0.9781	0.9985	0.9961	0.9838	0.9682
	20000	0.9781	0.9985	0.9961	0.9838	0.9682
	25000	0.9781	0.9985	0.9961	0.9838	0.9682

*0.5*	5	0.9744	0.9986	0.9957	0.9823	0.9653
	100	0.9703	0.9993	0.9958	0.9826	0.9659
	600	0.981	0.9986	0.9965	0.9855	0.9714
	1000	0.9794	0.9985	0.9962	0.9842	0.969
	20000	0.9781	0.9985	0.9961	0.9838	0.9682
	25000	0.9781	0.9985	0.9961	0.9838	0.9682

*1.0*	5	0.9707	0.997	0.9938	0.9745	0.9504
	100	0.953	0.9994	0.9938	0.9741	0.9495
	600	0.9831	0.9987	0.9968	0.9869	0.9742
	1000	0.918	0.9986	0.9889	0.9525	0.9094
	20000	0.9785	0.9985	0.9961	0.984	0.9686
	25000	0.9781	0.9985	0.9961	0.9838	0.9682

**Table 5:** The calculated segmentation evaluation metrics for level set method and proposed approach in BRATS Training data 2012.

Patient No.	Method	*TP*	*FP*	*TN*	*FN*	*Sensitivity*	*Specificity*	*Accuracy*	*Dice index*	*Jaccard*
Patient 1	Flair	1102	178	17090	66	0.9434	0.9896	0.9867	0.9	0.818
	*Thermal map*	*1142*	*18*	*17250*	*26*	*0.9777*	*0.9989*	*0.9976*	*0.9811*	*0.9629*

Patient 2	Flair	1067	165	15176	2	0.9981	0.9892	0.9898	0.9274	0.8646
	*Thermal map*	*1038*	*18*	*15323*	*31*	*0.971*	*0.9988*	*0.997*	*0.9769*	*0.9549*

Patient 3	Flair	2891	498	14358	4	0.9986	0.9664	0.9717	0.9201	0.852
	*Thermal map*	*2767*	*41*	*14815*	*128*	*0.9557*	*0.9972*	*0.9904*	*0.9703*	*0.9424*

Patient 4	Flair	1678	342	9386	4	0.9976	0.9648	0.9696	0.9065	0.829
	*Thermal map*	*1561*	*44*	*9684*	*121*	*0.928*	*0.9954*	*0.9855*	*0.9498*	*0.9044*

Patient 5	Flair	1810	191	17340	69	0.9632	0.9891	0.9866	0.9329	0.8743
	*Thermal map*	*1803*	*123*	*17408*	*76*	*0.9595*	*0.9929*	*0.9897*	*0.9477*	*0.9005*

Patient 6	Flair	1982	628	16415	1	0.9994	0.9631	0.9669	0.863	0.759
	*Thermal map*	*1916*	*32*	*17011*	*67*	*0.9662*	*0.9981*	*0.9947*	*0.9748*	*0.9508*

**Table 6:** The calculated segmentation evaluation metrics for level set method and proposed approach in BRATS Training data 2013.

Patient No.	Method	*TP*	*FP*	*TN*	*FN*	*Sensitivity*	*Specificity*	*Accuracy*	*Dice index*	*Jaccard*
Patient 1	Flair	2831	371	16731	106	0.9639	0.9783	0.9761	0.9223	0.8558
	*Thermal map*	*2803*	*71*	*17031*	*134*	*0.9543*	*0.9958*	*0.9897*	*0.9647*	*0.9318*

Patient 2	Flair	1378	331	13666	26	0.9814	0.9763	0.9768	0.8853	0.7942
	*Thermal map*	*1307*	*34*	*13963*	*97*	*0.9309*	*0.9975*	*0.9914*	*0.9522*	*0.9089*

Patient 3	Flair	1274	413	17336	10	0.9922	0.9767	0.9777	0.8576	0.7507
	*Thermal map*	*1236*	*8*	*17741*	*48*	*0.9626*	*0.9995*	*0.997*	*0.9778*	*0.9566*

Patient 4	Flair	1762	560	18243	1	0.9994	0.9702	0.9727	0.8626	0.7585
	*Thermal map*	*1660*	*33*	*18770*	*103*	*0.9415*	*0.9982*	*0.9933*	*0.9606*	*0.9242*

Patient 5	Flair	2015	807	13191	0	1	0.9423	0.9496	0.8331	0.714
	*Thermal map*	*1901*	*12*	*13986*	*114*	*0.9434*	*0.9991*	*0.9921*	*0.9679*	*0.9378*

Patient 6	Flair	1383	644	13522	0	1	0.9545	0.9585	0.8111	0.6822
	*Thermal map*	*1271*	*20*	*14146*	*112*	*0.91901*	*0.9985*	*0.9915*	*0.9506*	*0.9059*

**Table 7:** The percent of tumor and healthy areas differentiated by segmentation in thermal images only.

Data set	Patient No.	Reduced false positive rate (%)	Reduced false negative rate (%)
Galimzianova et al. [50]	Patient 1	0.16	1.89
	Patient 2	1.03	1.82
	Patient 3	0.12	2.32
	Patient 4	0	2.22

BRATS 2012	Patient 1	5.13	0.93
	Patient 2	0.18	0.95
	Patient 3	0	3.08
	Patient 4	0	3.13
	Patient 5	1.27	0.82
	Patient 6	0	3.49

BRATS 2013	Patient 1	2.24	1.84
	Patient 2	1.78	2.15
	Patient 3	0.77	2.28
	Patient 4	0	2.8
	Patient 5	0	5.67
	Patient 6	0	4.4

## Data Availability

The data used to support the findings of this study are included within the article.
